# Exploring the multifaceted challenges of gastrointestinal metastases in lung adenocarcinoma: a case report highlighting diagnostic dilemmas and therapeutic innovations

**DOI:** 10.3389/fonc.2024.1486371

**Published:** 2024-11-26

**Authors:** Xinxin Xu, Qian Zhou, Peng Chen, Chengzhou Du, Yonghua Huang, Xiaoxin Gao, Shumei Xu, Jinling Wu, Tianxiao He, Hongtao Li

**Affiliations:** ^1^ Department of General Surgery, The 940th Hospital of Joint Logistics Support Force of Chinese People’s Liberation Army, Lanzhou, Gansu, China; ^2^ Department of General Surgery, The Second Clinical Medical School of Lanzhou University, Lanzhou, Gansu, China; ^3^ Department of Radiology, The 940th Hospital of Joint Logistics Support Force of Chinese People’s Liberation Army, Lanzhou, Gansu, China

**Keywords:** lung cancer, gastrointestinal complications, metastasis, intestinal obstruction, immunotherapy response

## Abstract

**Background:**

Lung cancer, the primary cause of cancer-related deaths, often metastasizes early, commonly affecting the liver, brain, bones, and adrenal glands. Although gastrointestinal (GI) metastasis is rare, it poses significant diagnostic and therapeutic challenges and is frequently linked to severe complications.

**Case description:**

We present a case of a 61-year-old male with a history of lung cancer who presented with intestinal obstruction. The initial diagnosis of poorly differentiated adenocarcinoma was confirmed by computed tomography (CT) and bronchoscopic biopsy. The patient underwent chemotherapy, after which he developed intestinal obstruction. Further imaging and histopathological analysis indicated GI metastasis. Despite treatment with both chemotherapy and immunotherapy, the patient experienced recurrent obstruction, necessitating surgical intervention. Postoperatively, he had another episode of perforation, which was addressed with an emergency laparotomy, revealing metastatic adenocarcinoma at the site of perforation.

**Conclusions:**

This case highlights the complexities in diagnosing and treating GI metastases from lung cancer. It underscores the necessity for multimodal treatment strategies and underscores the urgent need for research focused on early detection methods to improve patient outcomes.

## 1 Introduction

Lung cancer is the leading cause of cancer-related mortality in men and ranks second in women globally (1). At the time of diagnosis, the majority of patients exhibit tumor metastasis, predominantly via lymphatic and hematogenous routes. The liver, brain, bone, and adrenal glands are common initial sites for metastatic spread of lung cancer (2, 3). In contrast, gastrointestinal (GI) metastasis from lung cancer is relatively infrequent and is typically identified in the context of severe GI complications such as perforation, hemorrhage, and intestinal obstruction (4). Early detection of GI metastasis is challenging due to its asymptomatic nature in the initial stages. Consequently, the investigation of surgical interventions and the elucidation of metastatic pathways associated with advanced GI metastasis are of paramount importance for enhancing patient survival. This study presents a case of non-small cell lung cancer (NSCLC) that required surgical intervention due to obstruction and perforation secondary to small intestine metastasis. We provide a comprehensive review of the patient’s diagnosis, therapeutic management, and metastatic profile.

## 2 Case report

A 61-year-old male was admitted to our facility presenting with intestinal obstruction. His medical history included a previous admission for hemoptysis at another hospital, where computed tomography (CT) and bronchoscopic lung biopsy confirmed NSCLC, specifically poorly differentiated adenocarcinoma, corroborated by immunohistochemical staining. The patient had received one cycle of chemotherapy with pemetrexed and cisplatin. Following chemotherapy, the patient presented with intestinal obstruction. Abdominal CT revealed focal thickening of the small intestine wall. Subsequently, a percutaneous biopsy of the small intestinal lesions was conducted. Histopathological analysis demonstrated the presence of poorly differentiated adenocarcinoma infiltrating the fibrous stroma, leading to the consideration of small intestinal metastasis from a primary lung malignancy. Consequently, the patient underwent a course of immunotherapy with Tislelizumab, during which he experienced recurrent episodes of incomplete intestinal obstruction.

**Figure 1:**
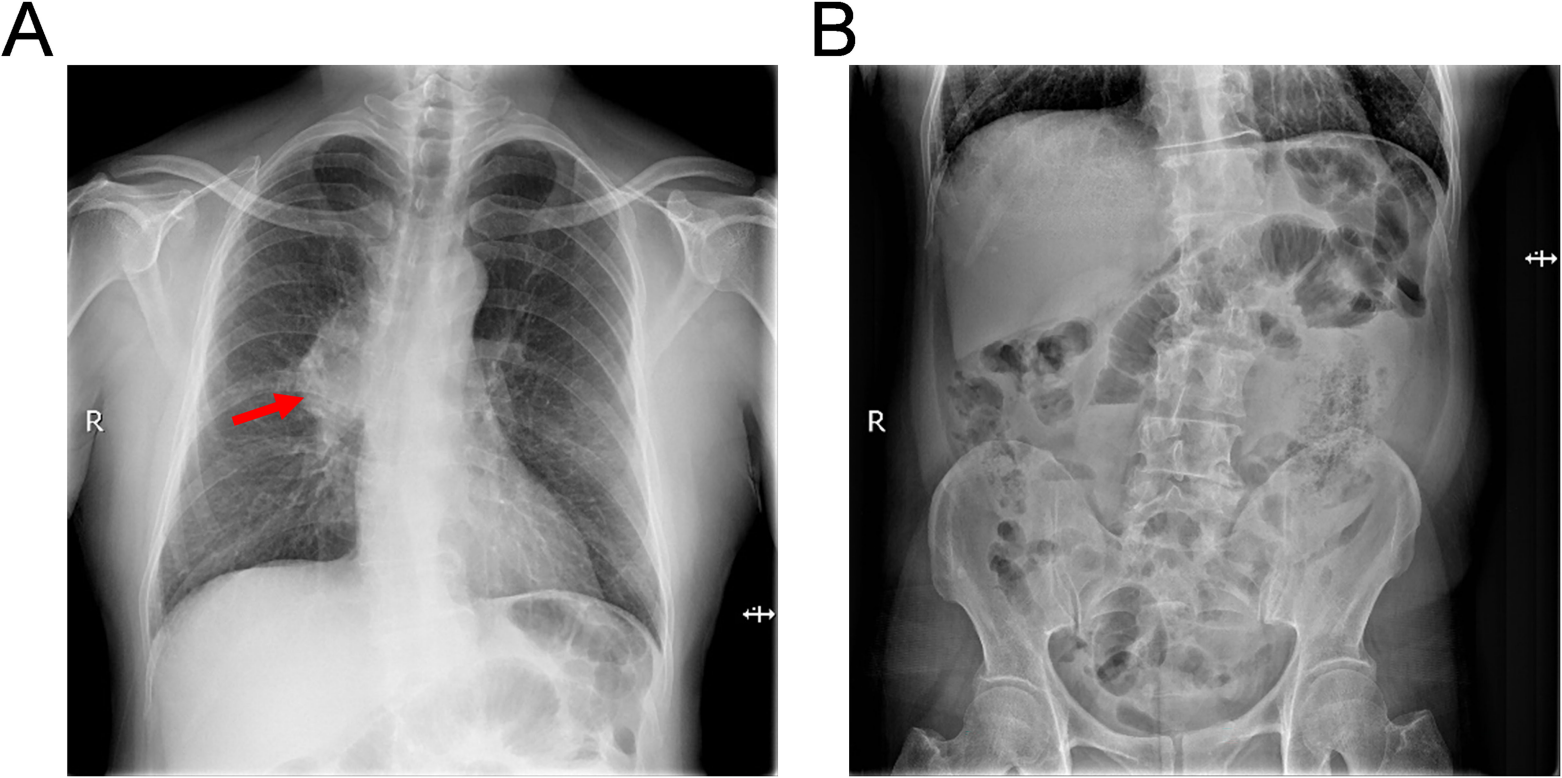
Radiographic assessment of thoracic and abdominal anatomy upon hospital presentation. **(A)** Thoracic radiography demonstrates bilateral pulmonary patchy opacities, indicative of infiltrative disease, along with pronounced broncho-vascular markings and a dense mass within the right pulmonary hilum (as shown by red arrows), suggesting a bronchial lung cancer or inflammatory process. **(B)** Abdominal radiography exhibits dilatation of intestine with the presence of air-fluid levels, consistent with the clinical suspicion of an incomplete intestinal obstruction.

**Figure 2:**
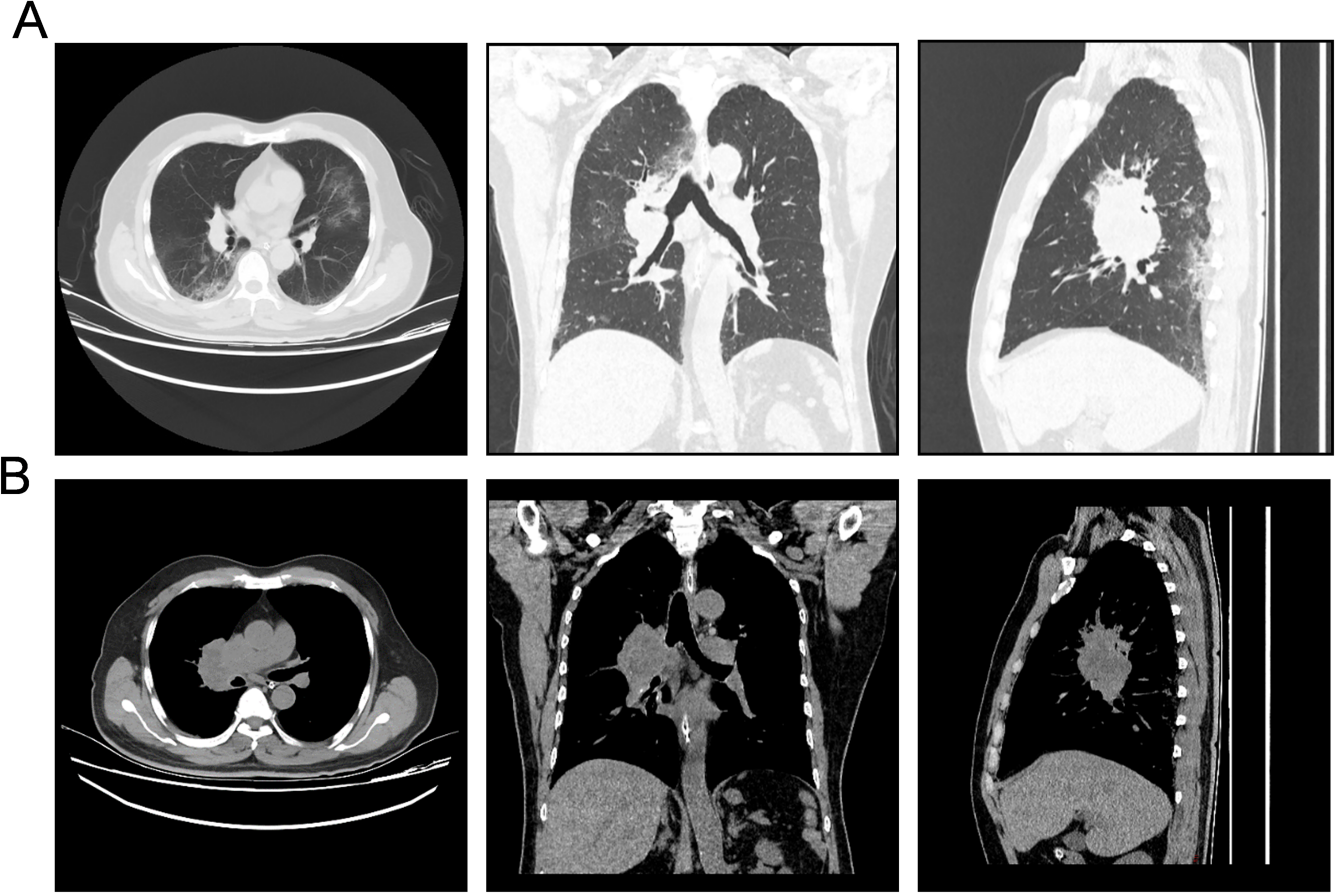
Computed tomography (CT) images depicting the invasive characteristics of the patient’s pulmonary neoplasm. **(A)** Pulmonary window CT images in axial, coronal, and sagittal planes (from left to right) illustrate the bronchial lung cancer’s infiltrative pattern and expansive growth within the lung parenchyma, delineating its borders and relationship to surrounding structures. **(B)** Mediastinal window CT images in axial, coronal, and sagittal planes (from left to right) demonstrate the extent of bronchial lung cancer’s invasion into the mediastinal structures, highlighting the impact on adjacent tissues and potential nodal involvement.

**Figure 3:**
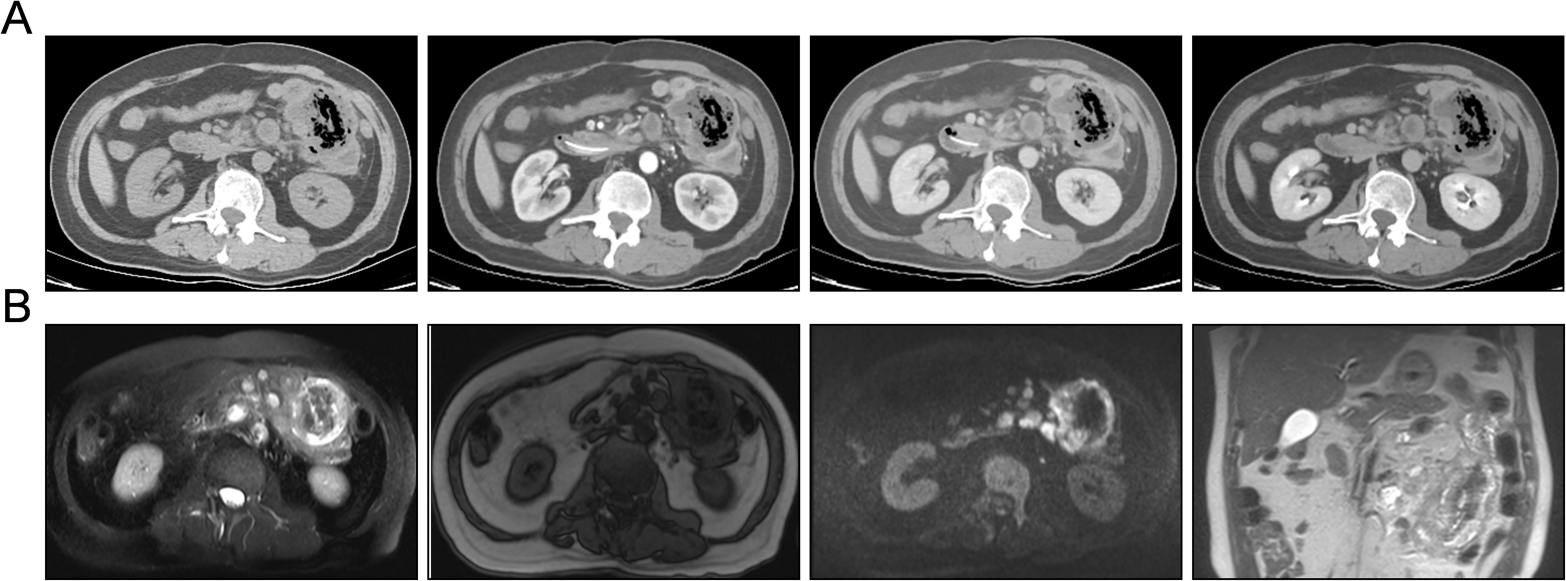
Contrast-enhanced CT and magnetic resonance imaging (MRI) demonstrating tumor metastasis within the small intestine. **(A)** Contrast-enhanced CT images in a sequential display of normal scan, arterial phase, venous phase, and delayed phase (from left to right) reveal the extent of metastatic infiltration and growth within the small intestine. These images provide a temporal assessment of the tumor’s vascularity and enhancement patterns. **(B)** Abdominal MRI in a series of axial T2-weighted, T1-weighted, diffusion-weighted imaging (DWI), and coronal views of T2-weighted image (from left to right) illustrate the invasive growth of small intestinal metastases. The MRI sequences offer a comprehensive evaluation of the tumor’s morphology, tissue characteristics, and potential involvement of adjacent structures.

**Figure 4:**
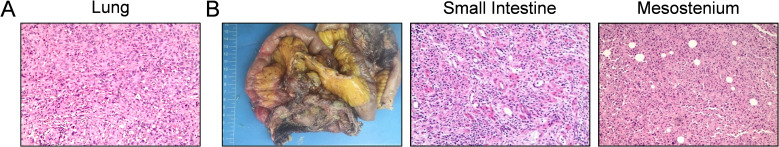
Histopathological examination of primary pulmonary and metastatic small intestinal lesions. **(A)** Hematoxylin and eosin (H&E) stained section of the primary lung tumor, exhibiting features characteristic of a poorly differentiated adenocarcinoma with atypia and loss of normal glandular architecture. **(B)** Sequential images from left to right: postoperative specimen of the small intestine with obstruction, H&E staining sections of the small intestine and mesentery. These images reveal metastatic poorly differentiated adenocarcinoma infiltrating the small intestinal mucosa and the mesenteric tissue, demonstrating the invasive nature of the malignancy.

Subsequently, the patient was referred to our center for persistent symptoms of incomplete intestinal obstruction, having fasted for over a month. Upon admission, a chest X-ray (**Figure 1**) revealed a dense mass in the right hemithorax, while an abdominal X-ray confirmed the clinical diagnosis of incomplete intestinal obstruction. A subsequent chest CT (**Figure 2**) confirmed the presence of centrally located lung cancer. Enhanced CT and MRI of the abdomen (**Figure 3**) depicted a circumferential small bowel abscess, indicative of obstruction, likely due to perforation from an intestinal metastatic tumor. The patient underwent exploratory laparotomy, intestinal resection and anastomosis to release intestinal obstruction. Postoperative pathology (**Figure 4**) confirmed metastatic lung cancer in a segment of the small intestine and adjacent lymph nodes. Immunohistochemical analysis demonstrated positivity for Cytokeratin 7 (CK7), Transcription Termination Factor 1 (TTF1) and SWI/SNF-related, matrix-associated, actin-dependent regulator of chromatin, subfamily A, member 4 (SMARCA4) in the metastatic small intestinal tumor.

On the fifth postoperative day, the patient presented with acute abdominal pain and peritoneal irritation, accompanied by the drainage of yellow-green fluid from the abdominal tube, indicative of intestinal content leakage. These symptoms necessitated an urgent second exploratory laparotomy. Intraoperative assessment revealed that the previously performed intestinal anastomosis had healed uneventfully, showing no signs of complications. However, a perforation was identified approximately 30 cm distal to the anastomosis within the small intestine. Histopathological examination of the perforated site post-surgery confirmed the presence of metastatic adenocarcinoma, suggesting a malignant perforation.

In light of the non-existence of clinically relevant genetic aberrations within the primary pulmonary tumors and the metastatic small intestinal lesions, coupled with the patient’s diminished tolerance for chemotherapy, the presence of a markedly elevated PD-L1 tumor proportion score (TPS) of approximately 95% warranted the implementation of a therapeutic strategy encompassing PD-1 monoclonal antibody monotherapy. This decision was endorsed following an exhaustive, multidisciplinary oncologic assessment. As of the latest follow-up, the patient has completed a duo of postoperative cycles of PD-1 immune checkpoint inhibitor therapy. Subsequent evaluative assessments confirmed the uneventful healing of the intestinal anastomosis. Radiological surveillance has documented a diminution in the dimensions of the primary pulmonary neoplasm, which may prognosticate a salutary clinical trajectory (**Supplementary Figure 1**).

## 3 Discussion

Adenocarcinoma is the predominant histological subtype of lung cancer, with GI metastases occurring in a mere 0.26% to 1.77% of cases in clinical settings (5, 6). However, post-mortem examinations reveal a higher incidence of abdominal metastasis, with rates ranging from 4.7% to 14% (3, 7, 8). This discrepancy underscores the underdiagnosis and limited understanding of GI metastases in lung cancer. Early symptoms of small bowel metastasis are subtle, often manifesting as weight loss, abdominal discomfort, bloating, and constipation. In advanced stages, perforation (42.0%), bleeding (24.6%), and intestinal obstruction (20.4%) are more prevalent, particularly in the jejunum (9). Given the prevalence of lung cancer, an increasing number of patients presenting with secondary abdominal symptoms due to metastatic disease can be anticipated, with an average survival time of less than 60 days post-diagnosis for these individuals.

Diagnosis of GI metastases is frequently delayed until the onset of severe complications such as intestinal obstruction or perforation, highlighting the importance of timely and accurate diagnostic efforts. Imaging and endoscopic modalities are instrumental in tumor detection and characterization. CT findings suggestive of GI metastasis include short segmental wall thickening, polypoid masses in the small intestine, associated local lymph node enlargement, perforation, or intussusception (10, 11). Capsule endoscopy can also visualize small intestinal lesions, although it has been reported to miss 16.7% of small intestinal tumors, especially those in the proximal jejunum (12), potentially contributing to the low rate of early detection. Positron Emission Tomography-Computed Tomography (PET-CT) has been demonstrated to be a valuable tool in the detection of subclinical GI metastases from lung cancer, as well as in the systematic assessment of extrapulmonary metastases and preliminary staging of small intestinal metastases from non-small cell lung cancer (13, 14).

Aggressive surgical management of small intestinal metastases may be considered for palliative care in select patients, particularly those with localized small intestinal involvement and limited digestive tract tumors (15). Post-surgical anti-tumor therapies, including immunotherapy or targeted therapy, are essential components of continued treatment. Our patient exhibited GI symptoms following one course of pemetrexed and cisplatin chemotherapy, and one course of Tislelizumab immunotherapy, suggesting a shrinkage of small intestinal metastases potentially leading to perforation. There have been reports of gastrointestinal perforation in NSCLC patients treated with dabrafenib and trametinib, with subsequent resolution of symptoms and favorable outcomes following targeted therapies post-palliative surgery (16). Mutations in genes such as TP53, LRP1B, and FGFR2 have been implicated in the pathogenesis and progression of poorly differentiated NSCLC and its small intestinal metastasis, offering insights for personalized treatment strategies through targeted therapies and radiation (17). The case also underscores potential avenues for future research, particularly the utility of immunotherapy in treating gastrointestinal metastases from lung adenocarcinoma. Further studies should investigate biomarkers predictive of immunotherapy response and explore combinatorial therapies to enhance treatment efficacy and personalize care for patients with lung cancer metastatic to the gastrointestinal tract.

The specific metastatic pathways of small intestinal metastasis from lung cancer remain elusive. The metastatic spread to the intraperitoneal region is thought to primarily involve hematogenous and lymphatic routes (18, 19). The role of tumor cell implantation and metastasis, particularly through the swallowing of sputum containing tumor cells, requires further elucidation, as does the mechanism by which tumor cells tolerate gastric acid (20). Our patient presented with multiple gastrointestinal metastases, and tumor cell characteristics were identified on sputum smears, supporting the hypothesis of sputum implantation metastasis. The underlying mechanisms of this phenomenon warrant further investigation.

## 4 Conclusion

In conclusion, our case report underscores the intricate nature and challenges associated with managing gastrointestinal metastases from lung adenocarcinoma, especially when complications such as intestinal obstruction arise. The patient’s journey through multimodal treatment, including chemotherapy and immunotherapy, and the subsequent need for surgical intervention due to recurrent obstruction and perforation, highlight the critical need for early detection and a personalized, multidisciplinary therapeutic strategy. This case also underscores the imperative for ongoing research to unravel the mechanisms behind metastasis and to devise more efficacious treatment options for patients with lung cancer that has metastasized to the gastrointestinal tract, ultimately aiming to improve clinical outcomes and patient quality of life.

## Data Availability

The original contributions presented in the study are included in the article/**Supplementary Material**. Further inquiries can be directed to the corresponding authors.

## Supplementary material

Supplementary Figure 1 Time plot of the patient since the onset of illness.
