# Supplementary figures and images for: Exploring the multifaceted challenges of gastrointestinal metastases in lung adenocarcinoma: a case report highlighting diagnostic dilemmas and therapeutic innovations
Source: Front Oncol. 2024 Nov 26;14:1486371. doi: 10.3389/fonc.2024.1486371 (PMC11628370; doi:10.3389/fonc.2024.1486371)

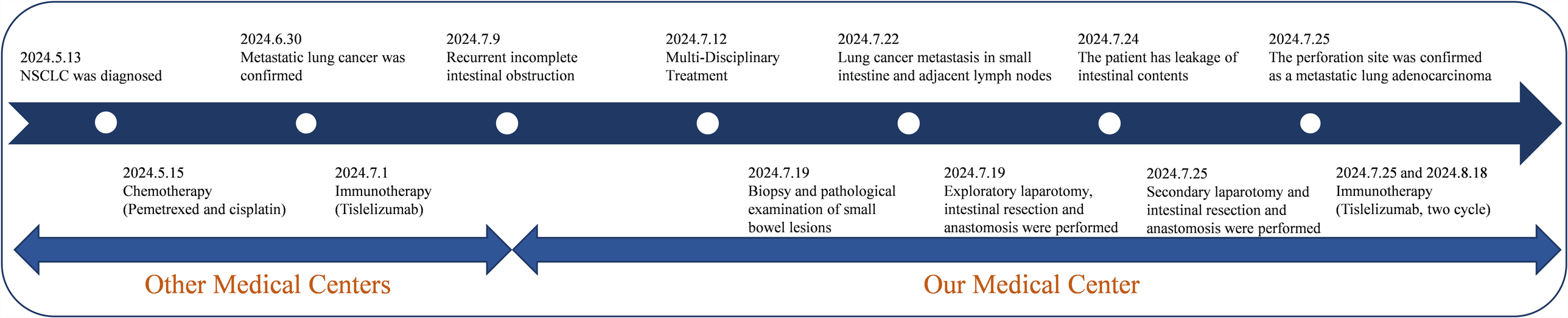

Supplement: Supplementary Figure 1 — Time plot of the patient since the onset of illness. [file Image1.tif]
